# A Comparison Study of Impulsiveness, Cognitive Function, and P300 Components Between Gamma-Hydroxybutyrate and Heroin-Addicted Patients: Preliminary Findings

**DOI:** 10.3389/fnhum.2022.835922

**Published:** 2022-04-20

**Authors:** Tingting Zeng, Shida Li, Li Wu, Zuxing Feng, Xinxin Fan, Jing Yuan, Xin Wang, Junyu Meng, Huan Ma, Guanyong Zeng, Chuanyuan Kang, Jianzhong Yang

**Affiliations:** ^1^Department of Psychiatry, The Second Affiliated Hospital of Kunming Medical University, Kunming, China; ^2^The Guangzhou Baiyun Psychological Hospital, Guangzhou, China; ^3^Department of Substance Use Disorders, The Psychiatry Hospital of Yunnan, Kunming, China; ^4^Department of Psychosomatic Medicine, Tongji University School of Medicine, Shanghai East Hospital, Shanghai, China

**Keywords:** gamma-hydroxybutyrate (GHB) addiction, heroin dependence, impulsiveness, cognitive function, P300 components

## Abstract

**Purpose:**

The aim of this study was to investigate and compare impulsiveness, negative emotion, cognitive function, and P300 components among gamma-hydroxybutyrate (GHB)-addicted patients, heroin-dependent patients, and methadone maintenance treatment (MMT) subjects.

**Methods:**

A total of 48 men including 17 GHB addicts, 16 heroin addicts, 15 MMT subjects, and 15 male mentally healthy controls (HC) were recruited. All subjects were evaluated for symptoms of depression, anxiety, impulsiveness, and cognitive function through the Patient Health Questionnaire (PHQ-9), the Generalized Anxiety Disorder 7-item (GAD-7), the Barratt Impulsiveness Scale version II (BIS-II), the Beijing version of the Montreal Cognitive Assessment (BJ-MoCA), the behavioral test (response time), and event-related potential P300 detection.

**Results:**

(1) The mean scores of BIS-II in the GHB addiction group, heroin dependence group, and MMT group were significantly higher than those of the HC group (*F* = 30.339, *P* = 0.000). (2) The total scores of BJ-MOCA in GHB addiction group was the worst among the four groups, followed by heroin addiction, MMT group and HC group (*F* = 27.880, *P* = 0.000). (3) The response time in the GHB addiction group was the longest among the four groups, followed by the heroin addiction, MMT, and HC groups (*F* = 150.499, *P* = 0.000). (4) The amplitude and latency of P300 in GHB addiction subjects were significantly lower and longer than those of the MMT group and the HC group. (5) For the three types of addiction, the P300 amplitudes at Fz, Cz, Pz, T5, and T6 were negatively correlated with the scores of GAD-7, PHQ-9, and BIS-II; the P300 latencies were positively correlated with the response time and negatively correlated with the scores of the BJ-MoCA.

**Conclusion:**

People with an addiction were likely to have increased impulsiveness. The cognitive function of the GHB and heroin-addicted subjects, including the heroin detoxification and the MMT groups, was severely impaired, especially for the GHB-addicted patients. The impairment manifested as abnormalities of BJ-MoCA, response time, and P300 components.

## Introduction

Substance use disorders (SUDs) bring a heavy burden on healthcare systems. Electroencephalography is a translatable technique, which enables understanding of the pathobiology of SUDs through the evaluation of various event-related potential components (Ramlakhan et al., 2018). Electrophysiological variables may represent sensitive biomarkers of vulnerability for SUDs (Campanella et al., 2014). Event-related potentials (ERPs) are averaged scalp electroencephalography responses time-locked to specific events during a sensory, motor, or cognitive task. As one component of ERP, P300 is a large, long-lasting component observed between 300 and 700 ms at central-parietal sites after the onset of visual or auditory stimuli (Habelt et al., 2020). P300 is associated with cognitive functions, including a wide range of attention, memory, and information processes (Campanella et al., 2014). Stimuli that would normally elicit P300 fail to do so when they are ignored or when attention is directed away from them (Duncan et al., 2009). Therefore, P300 is a sensitive measure of the capacity to allocate attentional resources (Johnson et al., 2004). The latency of P300 is sensitive to the time required for stimulus perception and classification, and its amplitude reflects the updating of representation in the working memory (Duncan et al., 2009).

In some studies, reduced amplitudes and increased latencies of the P300 components, compared to control subjects, have been found in patients suffering from alcohol use disorder (AUD) and heroin dependence, including those undergoing heroin detoxification and substitution treatment with methadone (Kreusch et al., 2014; Motlagh et al., 2018). Furthermore, a meta-analysis suggested that P300 amplitude reduction could be a useful vulnerable marker and a promising neurobiological endophenotype for SUD men (Euser et al., 2011).

Gamma-hydroxybutyrate (GHB) is an endogenous precursor of gamma-aminobutyric acid (GABA) and a central nervous system (CNS) depressant that has become notorious due to its abuse potential as a recreational drug in the form of a colorless, odorless liquid or white powder, tablet, or capsule under a number of street names (“Georgia Home Boy,” “Juice,” “Liquid Ecstasy,” “Mils,” “G,” “Liquid X,” “Liquid G,” and “Fantasy”) (Marinelli et al., 2020). Although it has been formally approved for the treatment of narcolepsy with cataplexy in adult patients in the United States and Europe and for the adjuvant treatment of alcohol dependence and withdrawal in Europe (Nicholson and Balster, 2001; Busardò et al., 2015), there are indications that the problematic use of GHB and its precursors, namely, gamma-butyrolactone (GBL) or 1,4-butanediol (1,4-BD), is on the rise (Brunt et al., 2014). GHB-intoxication often occurs during nonmedical use and generally results in a coma. GHB use can also lead to tolerance and dependence. After sudden cessation or reduction of intensive GHB use, a severe withdrawal syndrome may occur with symptoms varying from tremor, anxiety, and agitation to autonomic instability, hallucinations, and delirium (Van Noorden et al., 2010). Risky GHB use or GHB overdosing has been shown to result in unintended neurotoxic harm to memory and cognitive function (Van Amsterdam et al., 2012). Nonfatal overdoses and deaths related to GHB have also been reported (Karila et al., 2009). Therefore, GHB addiction can be considered a new form of SUD and has become a growing public health issue (Marinelli et al., 2020).

*In vivo* studies with urethane-anesthetized rats showed that the electrophysiological profile of GHB is close to typical drugs of abuse. Inhibition of nucleus accumbens (NAc) neurons and moderate to strong stimulation of dopaminergic (DA) transmission are distinctive features of diverse classes of abused drugs (Pistis et al., 2005). In previous electroencephalography (EEG) studies, GHB was shown to induce an electrophysiological pattern of “paradoxical EEG-behavioral dissociation” characterized by increased delta and theta oscillations that are usually associated with sleep during awake states (Jenney et al., 1962; Orioli et al., 1966). In a study of 19 healthy men, GHB increased current source density (CSD) of theta oscillations (5–7 Hz) in the posterior cingulate cortex (PCC) and alpha1 (8–10 Hz) oscillations in the anterior cingulate cortex; higher blood plasma values of GHB were associated with higher lagged phase synchronization (LPS) values of delta (2–4 Hz) oscillations between the PCC and the right inferior parietal lobules. Furthermore, GHB decreased global omega complexity (GOC) of alpha1 oscillations (von Rotz et al., 2017). These findings indicated that alterations in neuronal oscillations in the PCC mediated the psychotropic effects of GHB in healthy subjects (von Rotz et al., 2017). However, there are few studies of GHB addiction in clinical and ERP data. In order to improve prevention and treatment strategies for GHB addiction, it is necessary to explore its clinical manifestations, including cognitive function and ERP. It is also necessary to compare its characteristics with other SUDs, such as heroin dependence.

Therefore, in this study, we investigated and compared the impulsiveness, negative emotion, cognitive function, and P300 components among GHB addicts, heroin addicts, methadone maintenance treatment (MMT) subjects, and healthy controls (HCs). We hypothesized that there are severe cognitive deficits and impaired P300 components in GHB addicts, which are similar to heroin addicts. To the best of our knowledge, this is the first report addressing the comparison of GHB addiction, heroin dependence, and MMT.

## Materials and Methods

### Subjects

A total of 48 male addicts were recruited from the Guangzhou Baiyun Psychological Hospital. These addicts were currently using heroin or GHB, or they had used heroin in the past and were treated with methadone for the last 6 months. They had normal vision and hearing (or within the normal range after correction) and were right-handed.

We recruited 16 male heroin addicts meeting the *Diagnostic and Statistical Manual of Mental Disorders 5th edition* (DSM-5) criteria for Opioid Use Disorder, and 15 MMT subjects who had used only heroin in the past and who had undergone methadone maintenance treatment for a minimum of 6 months. The Opiate Withdrawal Scale (OWS) was used in heroin addicts, including patients undergoing heroin detoxification and substitution treatment with methadone (Bradley et al., 1987). In the OWS, a 4-point scale was used to rate each symptom/sign: absent (0), mild (1), moderate (2), and severe (3). The heroin addicts and the MMT subjects who scored between 0 and 1 on each subitem were included.

We recruited 17 GHB male addicts meeting the DSM-V for Other (or Unknown) Substance-Related Disorders who had used only GHB in the past. GHB withdrawal has many features similar to alcohol withdrawal, including tremor, sweating, anxiety, agitation, and confusion. Considering that there is no specific scale for assessing GHB withdrawal symptoms, we used a widely used scale for alcohol withdrawal–the Clinical Institute Withdrawal Assessment for Alcohol (CIWA-Ar) (Attilia et al., 2018), which is used clinically to monitor GHB/GBL withdrawal (Lingford-Hughes et al., 2016). Only GHB addicts with CIWA-Ar scores <9 were recruited in this study.

The exclusion criteria for the three addict groups were as follows: (1) diagnosis of other substance use disorders; (2) diagnosis of infectious disease; (3) history of head injury, neurological disorders, or loss of consciousness; (4) history of cardiovascular and cerebrovascular diseases and serious physical diseases; (5) having comorbid diagnoses of schizophrenia, intellectual disability, obsessive-compulsive disorder, and post-traumatic stress disorder; (6) in GHB addicts, CIWA-Ar scores ≥9; and (7) heroin dependents and MMT subjects who had withdrawal symptom ratings of moderate to severe on any subitem of the OWS.

A group of 15 mentally healthy HCs was recruited by advertisements in the local community. They had normal hearing and vision (or vision within the normal range after correction) and were right-handed. For the HC group, the exclusion criteria included (1) current or lifetime history of drug or alcohol abuse; (2) current or previous mental disorders (and/or treatment for it); (3) history of head injury, neurological disorders, or loss of consciousness; and (4) history of cardiovascular and cerebrovascular diseases and serious physical diseases.

This study was carried out in accordance with the latest version of the Declaration of Helsinki and was approved by the Ethics Committee of the Second Affiliated Hospital of Kunming Medical University. Participants' informed consent was obtained after the nature of the procedures had been fully explained. The registration number of this study is NTC 03910686.

### Measures

The Chinese version of the Patient Health Questionnaire-9 (PHQ-9), the Chinese version of the Generalized Anxiety Disorder 7-item (GAD-7), and the Chinese version of the Barratt Impulsiveness Scale version II (BIS-II).

The Chinese versions of scales with established reliability and validity were used to assess symptom severity, including PHQ-9 (Zhang et al., 2013) for depressive symptoms, GAD-7 for generalized anxiety symptoms (Spitzer et al., 2006; He et al., 2010), and BIS-II for impulsivity (Patton et al., 1995; Huang et al., 2013; Lu et al., 2013).

Symptom severity was defined as mild, moderate, moderately severe, or severe using the following cutoffs: scores of 5–9, 10–14, 15–19, and ≥20, respectively, on the PHQ-9 (Wang et al., 2014). Scores of 5, 10, and 15 were taken as the cutoffs for mild, moderate, and severe ranges on the GAD-7 (Wang et al., 2018).

The BIS-II is a self-report measure for assessing individual impulsive personality traits. The three basic factors of impulsiveness in BIS-II are attentional impulsiveness, motor impulsiveness, and non-planning impulsiveness (Patton et al., 1995). BIS-II was introduced to China in 2006 (Zhou et al., 2006). The Chinese version of BIS-II has 26 items and shows good reliability and validity (Zhou et al., 2006). Each item uses a 4-point scale: “never” is rated as 1, “occasionally” as 2, “often” as 3, and “always” as 4. The higher a subject's total score, the greater impulsivity they have.

### The Chinese Version of the Fagerstrom Test of Nicotine Dependence

The Chinese version of the Fagerstrom Test of Nicotine Dependence (FTND) with established reliability and validity was used to assess the degree of nicotine dependence. The scale consists of six items in total. The total score is used to evaluate nicotine dependence and ranges from 0 to 10. The higher the total score, the more severe the nicotine dependence (Niu et al., 2000; Huang et al., 2006).

### The OWS

The Opiate Withdrawal Scale was developed to evaluate the severity of opioid withdrawal symptoms (Bradley et al., 1987). It was introduced to China in the 1990s by the National Institute for Drug Dependence. The signs and symptoms related to opioid withdrawal are summarized into 32 items. A 4-point scale is used to rate each symptom/sign: zero (0), mild (1), moderate (2), and severe (3) (Zhang, 2005).

### The Clinical Institute Withdrawal Assessment for Alcohol (CIWA-Ar)

The Clinical Institute Withdrawal Assessment for Alcohol is a 10-item scale developed to monitor and rate the number and severity of symptoms occurring during the alcohol withdrawal syndrome (Attilia et al., 2018). Clinicians score each item on a Likert scale, and the maximum score is 67. The higher the score, the more severe the alcohol withdrawal syndrome (Higgins et al., 2019). CIWA-Ar scores of 0–8, 9–15, and 16 or more indicate mild, moderate, and severe withdrawal syndromes, respectively (Mayo-Smith, 1997). The CIWA-Ar was used clinically by Anne Lingford-Hughes et al. to monitor GHB/GBL withdrawal (Lingford-Hughes et al., 2016).

### The Beijing Version of the Montreal Cognitive Assessment

The Montreal Cognitive Assessment is a cognitive screening test used to detect mild cognitive impairment (MCI) (Nasreddine et al., 2005). The MoCA showed acceptable sensitivity (83.3%) and specificity (72.9%) for the identification of cognitive impairment in SUDs. It provides a time-efficient and resource-conscious way to identify patients with SUDs and neuropsychological impairment, thus addressing a critical need in the addiction treatment research community (Copersino et al., 2009).

The Beijing version of the Montreal Cognitive Assessment (BJ-MoCA) has been widely used in China for the screening of cognitive impairment. There are six dimensions, including visuospatial/executive, naming, attention, language, abstraction, memory, and orientation. The optimal cutoff point of the BJ-MoCA to detect cognitive impairment in the general population is a score of 26. A cutoff score of 25 is suggested if the subject had <12 years of education (Lu et al., 2011; Nie et al., 2012).

### ERP P300 Recording

#### Paradigm

An auditory oddball paradigm with 80% nontarget stimuli (65 db, 1,000 Hz) and 20% target stimuli (65 db, 1 200 Hz) presented binaurally through headphones in a pseudo-randomized order was used (duration of 500 ms with 10 ms rise and 10 ms fall time and an interstimulus interval of 700 ms). Subjects were seated with their eyes opened in a reclining chair and had to press a button with their dominant hand after target stimuli. All subjects were given practice tests to ensure that they could discriminate between target and nontarget tones.

#### Recording

Recording took place in a sound-attenuated and electrically shielded room. To reduce muscle artifacts in the EEG signal, the participants were instructed to sit in a comfortable position and avoid movement during recording. Considering the technical limitations and in order to reduce the complexity of the analysis, EEG signals from 17 electrodes—(F7/8, Fz, F3/4, T3/4, C3/4, Cz, T5/6, P3/4, Pz, and O1/2) according to the 10–20 standard of electrode placement with A1/2 (earlobes) as the reference—were recorded. Before data collection, the impedances of all the electrodes were monitored for each subject, to verify that its value was under 10 kΩ. Data were collected with a sampling rate of 512 Hz and an analogous bandpass filter (1–40 Hz). After channels were selected (F7/8, Fz, F3/4, T3/4, C3/4, Cz, T5/6, P3/4, Pz, and O1/2), data were averaged per condition (target vs. nontarget) and represented graphically in terms of latency (*x*-axis) and amplitude (*y*-axis). The P300 component was identified as the most positive component within the latency window of 250–600 ms. Peak latency was defined as the time point of maximum positive amplitude within the specific latency window. The 50 ms before and after the average peak latency interval for each group was selected as the time window. The amplitude corresponding to all time points in the window was averaged to obtain the average amplitude (Wei and Luo, 2010). EEG data were screened visually for artifacts, normal variants, and changes in alertness (the technician screening these data was blinded to group status). Only wave shapes based on at least 45 target trial (50% of the total) averages were accepted. The P300 amplitude and latency were determined using MATLAB software.

### Behavioral Test

A behavioral test based on the auditory oddball paradigm is described above. Subjects had to press the space bar with their right hand after the target stimuli. The average response time of their keystroke was recorded.

### Statistical Analysis

Statistical analyses were performed using SPSS 21.0 (Statistical Package for Social Sciences, IBM, Armonk, NY). Continuous data were presented as mean ± standard deviation (SD) or median [interquartile range]. Categorical data were presented as absolute numbers and percentages. The demographic and clinical characteristics of the four groups were compared using one-way ANOVA, Kruskal–Wallis H test, or Fisher's exact test. Kruskal–Wallis H test or analysis of covariance (ANCOVA) were used to compare the BIS-II scores, the BJ-MoCA scores, the response time, and the ERP data among the four groups with group as the fixed factor and age as a covariate. The amplitude and latency in Fz, Cz, Pz, T5, and T6 were correlated with the demographic data, the used substance, GAD-7, PHQ-9, BIS-II, BJ-MoCA, response time, and FTND scores of the three groups (i.e., heroin dependence, GHB addiction, and MMT groups). Multiple stepwise linear regression models were then used to evaluate the factors associated with the amplitude and latency in Fz, Cz, Pz, T5, and T6 positions. We used a two-sided α of 0.05 for statistical significance.

## Results

### Demographic and Clinical Characteristics Among the Four Groups

Notably, 17 GHB addicts, 16 heroin addicts, 15 MMT subjects, and 15 HCs were recruited in this study. As shown in Table 1, the average age of GHB addicts was 27.9 ± 5.3 years, which was significantly younger than the other three groups. Thus, in the analysis of BJ-MoCA, response time, BIS-11 and P300, age was used as a covariant to exclude the effect of age on cognitive differences. The mean duration of addiction in GHB addicts was 2.0 ± 1.4 years which was also the shortest duration among the three types of addiction. There were no statistical differences in marital status, employment status, years of education, and FTND scores among the four groups.

**Table 1:** Demographics and clinical characteristics of the four groups.

**Variable**	**GHB addiction**	**Heroin dependence**	**MMT group**	**Healthy controls**	***F*/*χ^2^/H***	***P* value**
	**(*n =* 17)**	**(*n =* 16)**	**(*n =* 15)**	**(*n =* 15)**		
Age	27.9 ± 5.3^a, c, d^	37.4 ± 6.2^b^	39.6 ± 6.2^b^	35.9 ± 6.2^b^	12.028	0.000^**^
Education	9 (9,12)	9 (9,9)	9 (9,12)	9 (9,12)	0.946	0.814
Marital status	6/11/0	3/11/2	1/13/1	3/11/1	5.696	0.419
Employment status	9/8	11/5	13/2	12/3	4.845	0.182
FTND	7.3 ± 1.3	7.1 ± 1.4	7.5 ± 1.1	6.5 ± 1.4	1.869	0.145
Duration of addiction	2.0 ± 1.4^c, d^	10.8 ± 5.8^b^	12.9 ± 5.9^b^	-	31.431	0.000^**^
GAD-7	3.6 ± 3.0	3.8 ± 3.1	3.9 ± 2.6	3.1 ± 2.8	0.240	0.868
PHQ-9	3.4 ± 2.7	3.6 ± 2.8	3.6 ± 2.8	3.3 ± 2.1	0.073	0.974
BIS-II	70.4 ± 1.9^a^	65.0 ± 1.7^a^	65.5 ± 1.8^a^	48.7 ± 1.7^b, c, d^	30.339	0.000^**^
BJ-MoCA	19.9 ± 0.5^a, c, d^	21.7 ± 0.5^a, b, d^	24.3 ± 0.5^a, b, c^	25.8 ± 0.5^b, c, d^	27.880	0.000^**^
Response time (ms)	491.0 ± 5.9^a, c, d^	467.5 ± 5.2^a, b, d^	421.7 ± 5.7^a, b, c^	340.9 ± 5.3^b, c, d^	150.499	0.000^**^

*Marital status, funmarried/married/divorced; Employment status, yes/no; FTND, Fagerstrom Test of Nicotine Dependence; GAD-7, Generalized Anxiety Disorder-7; PHQ-9, Patient Health Questionnaire-9; BIS-II, Barratt Impulsiveness Scale-II; BJ-MoCA, The Beijing version of Montreal Cognitive Assessment*.

** *p < 0.001*.

a *P < 0.05 compared to healthy controls*.

b *P < 0.05 compared to GHB addiction*.

c *P < 0.05 compared to heroin dependence*.

d *P < 0.05 compared to methadone treatment*.

The GAD-7 and PHQ-9 scores of the four groups showed no statistically significant difference. The mean scores of BIS-II in the GHB addiction group, heroin dependence group, and MMT group were significantly higher than that of the HC group (*F* = 30.339, *P* = 0.000).

### Comparison of BJ-MoCA Scores Among the Four Groups

As shown in Table 1, the total score of BJ-MoCA in GHB addiction group was 19.9 ± 0.5, which was the worst among the four groups, followed by heroin addiction, MMT group and HC group. The scores of visuospatial, executive function, and memory in the GHB addiction group and the heroin addiction group were lower than that of the HC group, but there was no significant difference between the MMT group and the HC group (*H* = 22.040, *P* = 0.000; *H* = 17.001, *P* = 0.001). The scores of attention in the GHB addiction group were lower than that of the MMT group and the HC group, but there was no significant difference between the heroin-dependent group and the GHB addiction group (*H* = 12.032, *P* = 0.007).

### Comparison of Response Time (ms) Among the Four Groups

As shown in Table 1, the response time in the GHB addiction group was the longest among the four groups, followed by the heroin addiction group, the MMT group, and the HC group (*F* = 150.499, *P* = 0.000).

### Mean Amplitude and Latency of P300 at Each Position in the Four Groups

**Figure 1:**
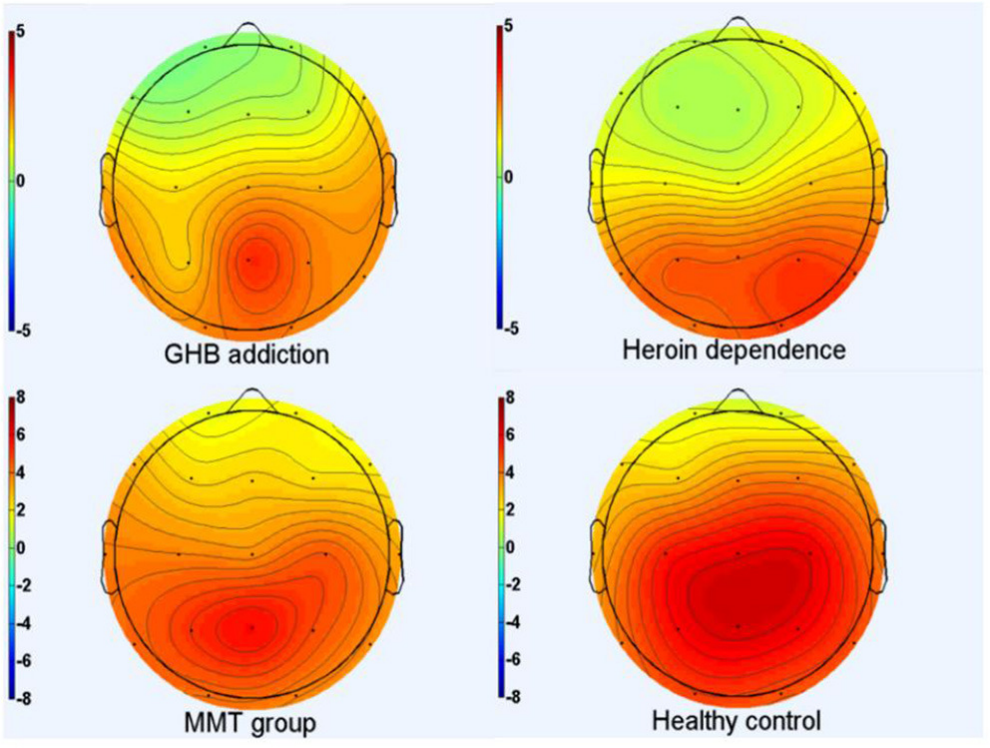
P300 topographic maps. Scalp topographic maps depicting mean P300 amplitudes around the mean latency ±50 milliseconds for target stimuli of GHB addiction, heroin dependence, MMT and HC group.

**Figure 2:**
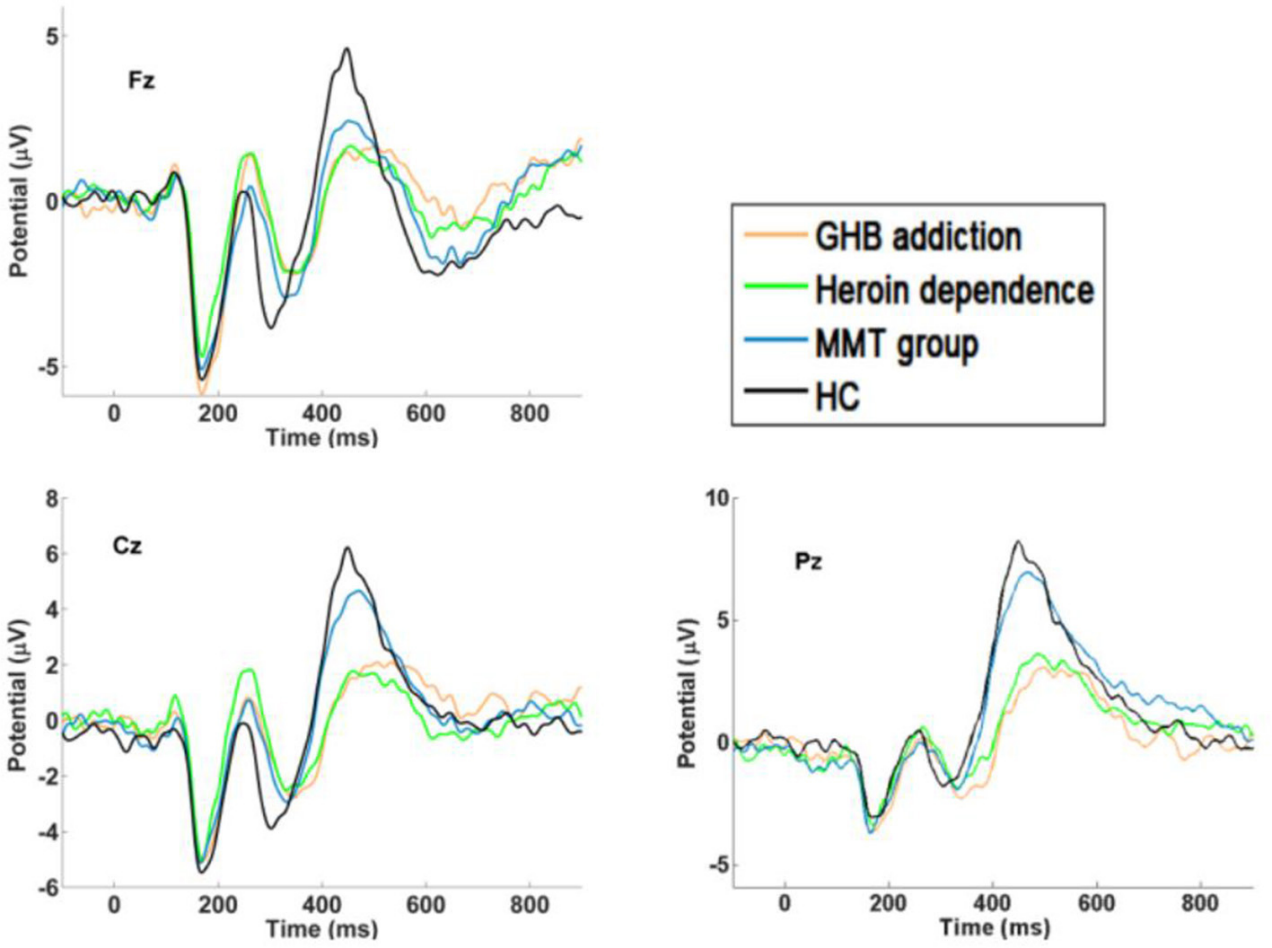
P300 waveforms. Difference waveforms for target stimuli of GHB addiction, heroin dependence, MMT and HC group.

**Figure 3:**
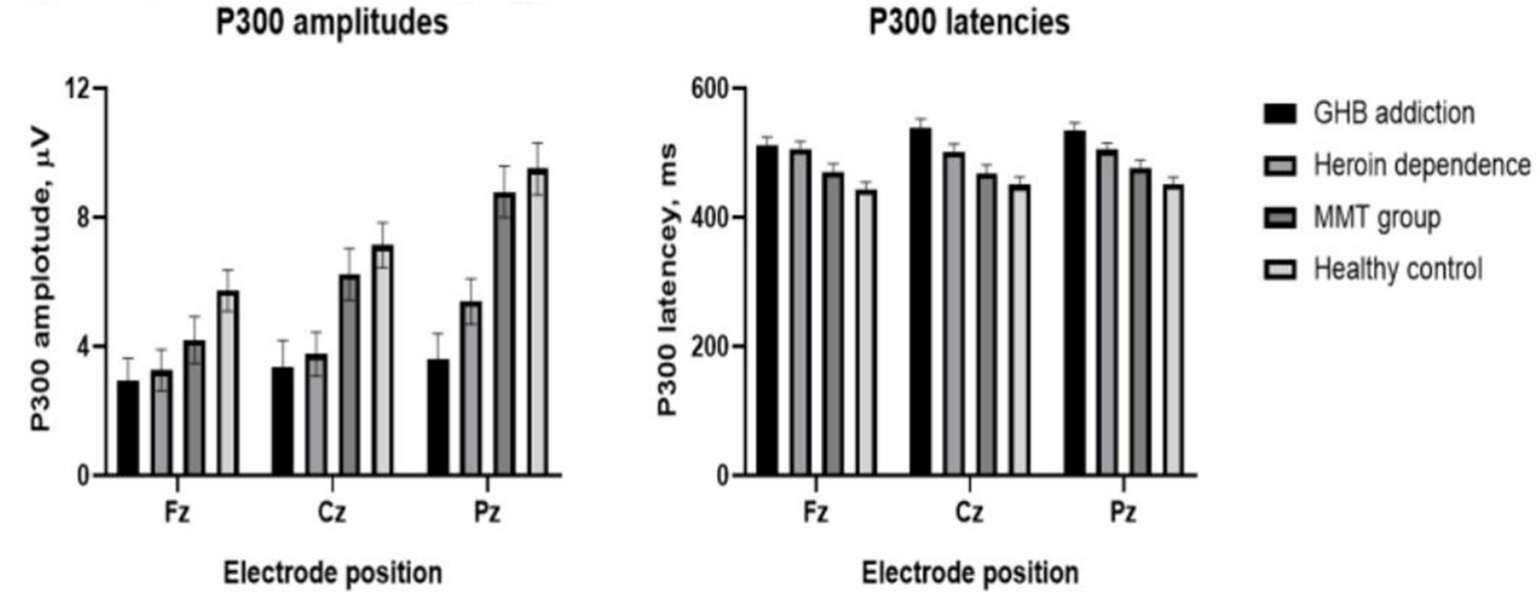
Means and standard deviation scores by groups. Group means and standard deviation (M ± SD) for P300 amplitudes (left); group means and standard deviation (M ± SD) for P300 latencies (right).

As shown in Figures 1–3 and Table 1, in the frontal lobe (F3, F4, F7, and Fz), central area (C3/4 and Cz), temporal lobe (T4, T5, and T6), parietal lobe (P3/4 and Pz), and occipital lobe (O1/2), the amplitude of P300 in GHB addiction subjects was significantly lower than that of the MMT group and the HC group. There was no significant difference in the amplitude of P300 between the GHB addiction group and the heroin addiction group. The latency of P300 in GHB addiction subjects was significantly longer than that of the MMT group and the HC group. In the temporal lobe (T4, T5, and T6), the latency of P300 in the GHB addiction group was significantly longer than that of the heroin addiction group. However, there was no significant difference between the GHB addiction group and the heroin addiction group for the latency of P300 in other brain regions.

### The Relationship of P300 Data With Clinical Data in the Three Addiction Groups

As shown in Tables 2–**4**, among the three addiction groups, P300 amplitudes at Fz, Cz, Pz, T5, and T6 were negatively correlated with the duration of addiction and scores of GAD-7, PHQ-9, and BIS-II. P300 latencies were negatively correlated with the score of BJ-MoCA but positively correlated with response time. In GHB addiction subjects, P300 latencies were positively correlated with the age of the subjects. P300 amplitudes were negatively correlated with the age of the heroin-dependent and MMT subjects.

**Table 2:** Stepwise regression model results for mean amplitude and mean latency of the GHB addiction.

**Variable**		**Adjusted R^**2**^**	**B**	**95% CI**	***t***	***P* value**
**Dependent**	**Independent**					
**GHB addiction**						
MA at Fz		0.631				
	BIS–II		−0.281	[−0.394, −0.169]	−5.324	0.000^**^
MA at Cz		0.222				
	GAD−7		−0.340	[−0.648, −0.033]	−2.362	0.032^*^
MA at Pz		0.624				
	Duration of addiction		−0.978	[−1.543, −0.412]	−3.707	0.002^*^
	PHQ−9		−0.463	[−0.758, −0.167]	−3.360	0.005^*^
MA at T5		0.279				
	Duration of addiction		−1.178	[−2.113, −0.242]	−2.682	0.017^*^
MA at T6		0.529				
	GAD−7		−0.702	[−1.045, −0.358]	−4.352	0.001^*^
ML at Fz		0.617				
	RT		3.066	[1.802, 4.329]	5.173	0.000^**^
ML at Cz		0.774				
	BJ–MoCA		−14.934	[−28.168, −1.701]	−2.420	0.030^*^
	RT		2.586	[0.948, 4.224]	3.385	0.004^*^
ML at Pz		0.605				
	RT		3.170	[1.831, 4.508]	5.047	0.000^**^
ML at T5		0.454				
	RT		2.797	[1.222, 4.373]	3.785	0.002^*^
ML at T6		0.556				
	RT		2.538	[1.358, 3.719]	4.585	0.000^**^

*CI, confidence interval; MA, mean amplitude; ML, mean latency; RT, response time*.

* *p < 0.05*,

** *p < 0.001*.

*BJ-MoCA, The Beijing version of Montreal Cognitive Assessment; GAD-7, Generalized Anxiety Disorder-7; PHQ-9, Patient Health Questionnaire-9; BIS-II, Barratt Impulsiveness Scale-II*.

### The Stepwise Regression Analysis of P300 Data With Clinical Data in the Three Addiction Groups

As shown in Table 2, in the GHB addiction group, multiple stepwise linear regression analysis found that P300 amplitudes at Fz, Cz, Pz, T5, and T6 were negatively correlated with the duration of the addiction and scores of GAD-7, PHQ-9, and BIS-II. P300 latencies were negatively correlated with the scores of BJ-MoCA but positively correlated with the response time.

As shown in Table 3, in the heroin addiction group, P300 amplitudes at Fz, Cz, Pz, T5, and T6 were negatively correlated with GAD-7, PHQ-9, BIS-II, scores, and response time and positively correlated with education and BJ-MoCA scores. P300 latencies were negatively correlated with BJ-MoCA scores but positively correlated with response time.

**Table 3:** Stepwise regression model results for mean amplitude and mean latency of the heroin dependence.

**Variable**		**Adjusted R^**2**^**	**B**	**95% CI**	***t***	***P* value**
**Dependent**	**Independent**					
**Heroin dependence**						
MA at Fz		0.586				
	RT		−0.087	[−0.127, −0.047]	−4.720	0.000^**^
MA at Cz		0.710				
	BIS–II		−0.624	[−0.913, −0.336]	−4.674	0.000^**^
	GAD−7		−0.758	[−1.043, −0.473]	−5.797	0.000^**^
MA at Pz		0.816				
	Education		0.428	[0.013, 0.842]	2.229	0.044^*^
	BJ–MoCA		0.619	[0.304, 0.934]	4.241	0.001^*^
MA at T5		0.623				
	GAD−7		−0.395	[−0.701,−0.090]	−2.800	0.015^*^
	BJ–MoCA		0.391	[0.071, 0.711]	2.642	0.020^*^
MA at T6		0.503				
	PHQ−9		−0.347	[−0.605, −0.089]	−2.905	0.012^*^
	RT		−0.036	[−0.069, −0.003]	−2.356	0.035^*^
ML at Cz		0.383				
	RT		1.415	[0.470, 2.360]	3.212	0.006^*^
ML at Pz		0.280				
	RT		0.942	[0.169, 1.714]	2.615	0.020^*^
ML at T5		0.305				
	RT		1.013	[0.224, 1.802]	2.754	0.016^*^
ML at T6		0.224				
	BJ–MoCA		−6.587	[−12.711, −0.462]	−2.307	0.037^*^

*CI, confidence interval; MA, mean amplitude; ML, mean latency; RT, response time*.

* *p < 0.05*,

** *p < 0.001*.

*BJ-MoCA, The Beijing version of Montreal Cognitive Assessment; GAD-7, Generalized Anxiety Disorder-7; PHQ-9, Patient Health Questionnaire-9; BIS-II, Barratt Impulsiveness Scale-II*.

As shown in Table 4, in the MMT group, the P300 amplitudes at Fz, Cz, Pz, T5, and T6 were negatively correlated with GAD-7, PHQ-9, and BIS-II scores but positively correlated with BJ-MoCA scores. P300 latencies were correlated negatively with BJ-MoCA scores but positively correlated with response time and BIS-II scores.

**Table 4:** Stepwise regression model results for mean amplitude and mean latency of the MMT group.

**Variable**		**Adjusted R^**2**^**	**B**	**95% CI**	***t***	***P* value**
**Dependent**	**Independent**					
**MMT group**						
MA at Fz		0.528				
	GAD−7		−0.806	[−1.232, −0.380]	−4.084	0.001^*^
MA at Cz		0.650				
	BJ–MoCA		1.574	[0.919, 2.228]	5.193	0.000^**^
MA at Pz		0.855				
	BIS–II		−0.453	[−0.560, −0.346]	−9.126	0.000^**^
MA at T5		0.675				
	PHQ−9		−0.894	[−1.246, −0.542]	−5.487	0.000^**^
MA at T6		0.294				
	PHQ−9		−0.746	[−1.361, −0.130]	−2.616	0.021^*^
ML at Pz		0.246				
	BIS–II		2.777	[0.236, 5.319]	2.361	0.035^*^
ML at T5		0.304				
	RT		0.879	[0.168, 1.590]	2.670	0.019^*^
ML at T6		0.372				
	BJ–MoCA		−11.113	[−18.995, −3.231]	−3.046	0.009^*^

*CI, confidence interval; MA, mean amplitude; ML, mean latency; RT, response time; MMT, methadone maintenance treatment*.

* *p < 0.05*,

** *p < 0.001*.

*BJ-MoCA, The Beijing version of Montreal Cognitive Assessment; GAD-7, Generalized Anxiety Disorder-7; PHQ-9, Patient Health Questionnaire-9; BIS-II, Barratt Impulsiveness Scale-II*.

### Statistical Power

Given the small sample size, based on research purposes, we calculated the statistical power of BIS-II, MoCA, reaction time, and P300. The statistical power was 100%, 99%, 100%, and 85%, respectively.

## Discussion

In this study, the mean age of the GHB addiction group was the youngest among the three addictive groups, suggesting that as a recreational drug, GHB could become an addictive substance for young people. In a systematic review, people using GHB recruited from the general population were predominantly young men (median 74%, range: 47–90%), with a mean age of 27 years (range: 24–32 years) (Dijkstra et al., 2021). The mean scores of BIS-II in the GHB addiction, heroin addiction, and MMT groups were significantly higher than those of HCs. The multiple stepwise linear regression analysis showed that the BIS-II scores were negatively correlated with the amplitude of P300 in all groups of GHB addiction, heroin addiction, and MMT, suggesting that people with an addiction were likely to have increased impulsiveness; and higher impulsivity could affect cognitive processing, such as working memory, as indicated by the meaning of amplitude of P300. Impulsivity, the tendency to act without sufficient consideration of potential consequences in pursuit of short-term rewards, is a vulnerability marker for SUDs (Verdejo-Garcia and Albein-Urios, 2021). The cognitive processes affected by higher impulsivity included attention, reflection, inhibition, and choices involving risk and reward (Sharma et al., 2014; Vassileva and Conrod, 2019). There is rich evidence about the role of impulsivity in SUDs and addictive disorders (D'Amour-Horvat et al., 2021; Ehlers et al., 2021). A recent study also found that treatment adherence is inversely related to impulsive behaviors (López-Torrecillas et al., 2021). Therefore, high impulsivity is an important aspect that must be considered in the prevention and treatment of GHB addiction and heroin addiction.

For the cognitive function, the total scores of BJ-MoCA and the response time in GHB addiction group were the worst among the four groups, followed by heroin addiction, then MMT group and HC group. The mean age was the youngest in the GBH addiction group, and the mean duration of addiction in this group was 2.0 ± 1.4 years, which was the shortest duration among the three types of substance addiction, suggesting that GHB addiction could severely impair cognitive function, and the cognitive impairment was even worse than heroin dependence for the same level of education. Furthermore, the cognitive dysfunction mainly consisted of the impairment of visual space, executive function, memory, and attention. GHB disrupted the acquisition of spatial learning and memory in adolescent rats (Sircar et al., 2011). The rats administered GHB displayed an impaired performance in the water maze test as compared to controls. Some significant alterations in density and functionality of γ-aminobutyric acid subunit B (GABA-B) and insulin-like growth factor-1 (IGF-1) receptors were observed in several brain regions associated with cognitive functions, e.g., the hippocampus (Johansson et al., 2014). In clinical data, multiple GHB-induced comas were associated with alterations of memory performance (Raposo Pereira et al., 2018), microstructural alterations in the white matter, and higher self-reported impulsivity (Raposo Pereira et al., 2020). In addition, among the influencing factors of cognitive function, age might have a certain effect (Li and Hsu, 2015). However, we neither observed a relationship between age and response time nor did we observe a relationship between age and the MoCA scores in this study.

The cognitive function of subjects in the MMT group, when compared to the heroin addiction group, was closely similar to that of the healthy control group. The results suggest that for GHB and heroin addicts, including heroin detoxification and substitution treatment by methadone, cognitive function was impaired when compared to healthy subjects. Even for the stable period after methadone treatment, the cognitive function of heroin addicts remained impaired. In a study by Bernhard W Müller et al. (2007), the amplitudes and latencies were not reduced in methadone-substituted opiate addicts for 19.2 months when compared to controls. This suggests that after receiving long-term methadone treatment, the cognitive function of former heroin addicts could recover and approach that of the control group. The longer the methadone substitution treatment, the better effects for the cognition and ERP for the heroin addiction group.

In the frontal lobe (F3, F4, F7, and Fz), central area (C3/4 and Cz), temporal lobe (T4, T5, and T6), parietal lobe (P3/4 and Pz), and occipital lobe (O1/2), the amplitude of P300 in GHB-addicted subjects was significantly lower than that of the MMT group and the control group, and there was no significant difference in the amplitude of P300 between the GHB-addicted group and the heroin-addicted group. The latency of P300 in GHB-addicted subjects was significantly longer than that of the MMT group and the control group, and there was no significant difference for the latency of P300 between the GHB-addicted group and the heroin-addicted group. The results of P300 components were consistent with the results of MoCA and response time, and multiple stepwise linear regression analysis demonstrated that the latency of P300 was negatively correlated with the MoCAa scores that were positively correlated with the response time, suggesting that the addiction could impair cognitive function, including information processing speed, especially for GHB addicts, which could cause severe cognitive dysfunction even for a relatively short duration of addiction. Given there is no similar research thus far, more research is needed to verify these results.

Multiple stepwise linear regression analysis also found that depression and anxiety were negatively correlated with the amplitude of P300 in the GHB addicts, heroin addicts, and MMT group, suggesting that negative emotion would increase the risk of cognitive dysfunction for the addicted subjects. The mood–cognition relationship was identified in different conditions, such as heroin users on MMT (Lin et al., 2012), dementia and mild cognitive impairment (Orgeta et al., 2015), and multiple sclerosis (Leavitt et al., 2020). In recent research, executive function impairments were suggested as likely contributory factors in the maintenance of affective disorders (Warren et al., 2020).

This study has a number of limitations. First, the generalization of the findings is limited by the sample size, though the statistical power was acceptable. The recruitment period lasted for 7 months, from 1 January 2021 to 31 July 2021 because the recruitment was disrupted by the COVID-19 pandemic. Therefore, the results warrant replication in a larger sample. Second, there was no comparison of the intervention for the GHB addiction group. It is difficult to have a unified treatment group given the fact that there is limited experience in the treatment of GHB addiction in China, and there is no specific effective treatment so far. Third, no analysis and comparison of other ERP components were performed. In the future, other components, such as N170 and N2, are needed to further explore the relationship with cognitive function. Fourth, no data for female addicts were collected. During the recruitment, only one female GHB addict in a withdrawal state was hospitalized, and she was excluded because she could not complete all the evaluations. Therefore, to match the composition of the GHB group, only male addicts were recruited in the other groups. Meanwhile, people using GHB presenting at the treatment unit were also mostly young men (50–89%, average age 27–34 years) (Dijkstra et al., 2021). The age distribution of GHB users might also have influenced our sample collection. Finally, the treatment duration of the MMT group should be extended in order to observe the long-term effects of treatment on cognition and ERP.

In conclusion, this study revealed that people with an addiction were likely to have higher impulsiveness, which could affect cognitive processing. The cognitive functions of the GHB addicts and heroin addicts, including those on heroin detoxification and MMT, were severely impaired, especially for GHB addicts, which were manifested as abnormalities of MoC, response time, and P300 components.

## Data Availability Statement

The raw data supporting the conclusions of this article will be made available by the authors, without undue reservation.

## Ethics Statement

The studies involving human participants were reviewed and approved by the Ethics Committee of the Second Affiliated Hospital of Kunming Medical University. The patients/participants provided their written informed consent to participate in this study. Written informed consent was obtained from the individual(s) for the publication of any potentially identifiable images or data included in this article.

## Author Contributions

JY, CK, and GZ contributed to project design, data analysis, data interpretation, and manuscript preparation. TZ, SL, LW, ZF, JY, XW, JM, and HM collected the data. XF conducted statistical analyses. All authors contributed to the article and approved the submitted version.

## Funding

This work was supported by National Key R&D Program of China Grants 2018YFC1314400 and 2018YFC1314405.

## Conflict of Interest

The authors declare that the research was conducted in the absence of any commercial or financial relationships that could be construed as a potential conflict of interest.

## Publisher's Note

All claims expressed in this article are solely those of the authors and do not necessarily represent those of their affiliated organizations, or those of the publisher, the editors and the reviewers. Any product that may be evaluated in this article, or claim that may be made by its manufacturer, is not guaranteed or endorsed by the publisher.
