# Supplementary material for: A Comparison Study of Impulsiveness, Cognitive Function, and P300 Components Between Gamma-Hydroxybutyrate and Heroin-Addicted Patients: Preliminary Findings
Source: Front Hum Neurosci. 2022 Apr 20;16:835922. doi: 10.3389/fnhum.2022.835922 (PMC9067320; doi:10.3389/fnhum.2022.835922)
Supplement: Supplementary file 1 [file Table_1.docx]

**Supplementary**

**Table 1 Mean amplitude and latency of P300 at each lead**

| Variable |  | GHB  addiction  (n=17) | Heroin dependence  (n=16) | MMT  group  (n=15) | Healthy  Controls  (n=15) | *F/H* | *P* value |
| --- | --- | --- | --- | --- | --- | --- | --- |
| MA | F3 | 2.5±0.8^a^ | 1.8±0.8^a,d^ | 3.9±0.8^c^ | 5.0±0.8^b,c^ | 3.633 | 0.018^*^ |
|  | F4 | 2.1±0.8^a^ | 1.6±0.7^a^ | 3.4±0.8 | 5.1±0.7^b,c^ | 4.412 | 0.007^*^ |
|  | F7 | 2.0±0.9^a^ | 2.7±0.8 | 4.6±0.8 | 4.9±0.8^b^ | 2.874 | 0.044^*^ |
|  | F8 | 1.5±0.8 | 2.5±0.7 | 4.3±0.8 | 3.5±0.7 | 2.168 | 0.102 |
|  | Fz | 3.0±0.7^a^ | 3.4±0.6^a^ | 4.4±0.7 | 5.7±0.6^b,c^ | 3.381 | 0.024^*^ |
|  | C3 | 2.5±1.0^a,d^ | 3.6±0.8^a,d^ | 6.4±0.9^b,c^ | 7.0±0.9^b,c^ | 5.558 | 0.002^*^ |
|  | C4 | 3.1±0.9^a,d^ | 4.1±0.8^a,d^ | 6.7±0.9^b,c^ | 6.9±0.8^b,c^ | 4.485 | 0.007^*^ |
|  | Cz | 3.4±0.8 | 3.8±0.7^a^ | 6.3±0.8 | 7.2±0.7^c^ | 10.865 | 0.012^*^ |
|  | T3 | 2.3±1.0 | 2.4±0.8 | 5.0±0.9 | 4.6±0.9 | 2.487 | 0.069 |
|  | T4 | 3.6±0.8^a^ | 3.4±0.7^a^ | 4.5±0.7 | 6.3±0.7^b,c^ | 4.726 | 0.005^*^ |
|  | T5 | 3.7±0.8^a,d^ | 4.4±0.8^a,d^ | 7.4±0.8^b,c^ | 6.9±0.8^b,c^ | 4.888 | 0.004^*^ |
|  | T6 | 3.8±0.8^a^ | 3.2±0.7^a,d^ | 5.3±0.8^c^ | 6.1±0.7^b,c^ | 3.314 | 0.026^*^ |
|  | P3 | 4.0±0.9^a,d^ | 5.1±0.8^a,d^ | 9.1±0.9^b,c^ | 8.6±0.8^b,c^ | 7.793 | 0.000^**^ |
|  | P4 | 4.1±0.9^a^ | 5.2±0.8^a^ | 6.3±0.8 | 7.4±0.8^b,c^ | 2.914 | 0.042^*^ |
|  | Pz | 3.6±0.8^a,d^ | 5.4±0.7^a,d^ | 8.8±0.8^b,c^ | 9.5±0.8^b,c^ | 11.526 | 0.000 ^**^ |
|  | O1 | 4.5±0.9^a^ | 3.7±0.8^a,d^ | 6.7±0.9^c^ | 7.1±0.8^b,c^ | 4.117 | 0.010 ^*^ |
|  | O2 | 4.6±0.7^a^ | 4.5±0.6^a^ | 5.4±0.7 | 6.9±0.6^b,c^ | 3.297 | 0.027^*^ |
| ML | F3 | 517.9±13.1^a,d^ | 498.0±11.7^a^ | 471.2±12.6^b^ | 445.9±11.8^b,c^ | 6.342 | 0.001^*^ |
|  | F4 | 518.6±11.7^a,d^ | 507.8±10.4^a,d^ | 471.6±11.2^b,c^ | 446.0±10.5^b,c^ | 9.418 | 0.000^**^ |
|  | F7 | 525.3±12.7^a,d^ | 491.1±11.3^a^ | 473.2±12.2^b^ | 448.8±11.4^b,c^ | 6.735 | 0.001^*^ |
|  | F8 | 517.9±12.5^a,d^ | 501.5±11.1^a^ | 470.8±12.0^b^ | 457.0±11.2^b,c^ | 5.402 | 0.002^*^ |
|  | Fz | 511.3±13.2^a^ | 505.3±11.8^a,d^ | 471.3±12.7^c^ | 442.6±11.9^b,c^ | 6.866 | 0.000^**^ |
|  | C3 | 541.9±13.5^a^ | 494.0±12.0^a^ | 479.8±13.0 | 452.0±12.1^b,c^ | 14.231 | 0.003^*^ |
|  | C4 | 536.6±12.0^a^ | 492.8±10.7^a^ | 478.7±11.6 | 452.4±10.8^b,c^ | 14.298 | 0.003^*^ |
|  | Cz | 539.1±13.7^a,d^ | 502.8±12.2^a,d^ | 468.0±13.2^b,c^ | 450.3±12.3^b,c^ | 8.491 | 0.000^**^ |
|  | T3 | 539.9±12.3^a^ | 488.0±10.9 | 485.3±11.8 | 458.4±11.0^b^ | 15.070 | 0.002^*^ |
|  | T4 | 529.9±12.5^a,c,d^ | 490.5±11.1^a,b^ | 488.5±12.0^b^ | 458.9±11.3^b,c^ | 5.756 | 0.002^*^ |
|  | T5 | 541.9±11.8^a,c,d^ | 500.4±10.4^a,b^ | 483.9±11.3^b^ | 454.5±10.6^b,c^ | 10.126 | 0.000^**^ |
|  | T6 | 532.3±10.8^a,c,d^ | 500.6±9.6^a,b^ | 487.7±10.4^b^ | 459.8±9.7^b,c^ | 8.360 | 0.000^**^ |
|  | P3 | 534.8±12.1^a^ | 499.6±10.8^a^ | 481.5±11.7 | 455.8±10.9^b,c^ | 13.969 | 0.003^*^ |
|  | P4 | 537.8±12.4^a,d^ | 503.4±11.0^a^ | 472.5±11.9^b^ | 457.5±11.1^b,c^ | 8.436 | 0.000^**^ |
|  | Pz | 534.9±12.0^a^ | 505.0±11.6^a^ | 477.9±11.5 | 451.1±10.8^b,c^ | 18.393 | 0.000^**^ |
|  | O1 | 522.3±11.8^a^ | 497.0±10.4^a^ | 478.0±11.3 | 446.5±10.6^b,c^ | 18.370 | 0.000^**^ |
|  | O2 | 527.8±11.4^a^ | 509.5±10.1^a^ | 486.7±10.9 | 457.5±10.2^b,c^ | 17.262 | 0.001^*^ |

*Note*: MA=mean amplitude, ML=mean latency, MMT=methadone maintenance treatment. **p*<0.05, ***p*<0.001. ^a^*P*<0.05 compared to healthy controls. ^b^*P*<0.05 compared to GHB addiction. ^c^ *P*<0.05 compared to heroin dependence. ^d^*P*<0.05 compared to methadone treatment.

**Table 2 Correlation analysis of** **mean amplitude and mean latency in GHB addiction**

| Variable | | *r* | *P* value |
| --- | --- | --- | --- |
| Dependent | Independent |  |  |
| GHB addiction |  |  |  |
| MA at Fz |  |  |  |
|  | Duration of addiction | -0.581 | 0.014^*^ |
|  | GAD-7 | -0.521 | 0.032^*^ |
|  | BIS-II | -0.809 | 0.000^**^ |
|  | RT | -0.509 | 0.037^*^ |
| MA at Cz |  |  |  |
|  | Duration of addiction | -0.513 | 0.035^*^ |
|  | GAD-7 | -0.521 | 0.032^*^ |
| MA at Pz |  |  |  |
|  | Duration of addiction | -0.637 | 0.006^*^ |
|  | PHQ-9 | -0.590 | 0.013^*^ |
| MA at T5 |  |  |  |
|  | Duration of addiction | -0.569 | 0.017^*^ |
| MA at T6 |  |  |  |
|  | Duration of addiction | -0.556 | 0.020^*^ |
|  | GAD-7 | -0.747 | 0.001^*^ |
| ML at Fz |  |  |  |
|  | BJ-MoCA | -0.601 | 0.011^*^ |
|  | RT | 0.801 | 0.000^**^ |
| ML at Cz |  |  |  |
|  | Age | 0.533 | 0.028^*^ |
|  | BJ-MoCA | -0.800 | 0.000^**^ |
|  | RT | 0.848 | 0.000^**^ |
| ML at Pz |  |  |  |
|  | Age | 0.541 | 0.025^*^ |
|  | BJ-MoCA | -0.609 | 0.009^*^ |
|  | RT | 0.793 | 0.000^**^ |
| ML at T5 |  |  |  |
|  | BJ-MoCA | -0.569 | 0.017^*^ |
|  | RT | 0.699 | 0.002^*^ |
| ML at T6 |  |  |  |
|  | Age | 0.501 | 0.041^*^ |
|  | BJ-MoCA | -0.554 | 0.021^*^ |
|  | RT | 0.764 | 0.000^**^ |

*Note*: MA=mean amplitude, ML=mean latency, RT=response time, *p<0.05, **p<0.001. BJ-MoCA: The Beijing version of Montreal Cognitive Assessment. GAD-7: Generalized Anxiety Disorder-7. PHQ-9: Patient Health Questionnaire-9. BIS-II: Barratt Impulsiveness Scale-II. FTND: Fagerstrom Test of Nicotine Dependence.

**Table 3 Correlation analysis of** **mean amplitude and mean latency in heroin dependence**

| Variable | | *r* | *P* value |
| --- | --- | --- | --- |
| Dependent | Independent |  |  |
| Heroin dependence |  |  |  |
| MA at Fz |  |  |  |
|  | Age | -0.603 | 0.014^*^ |
|  | BIS-II | -0.637 | 0.008^*^ |
|  | RT | -0.784 | 0.000^**^ |
| MA at Cz |  |  |  |
|  | Duration of addiction | -0.629 | 0.009^*^ |
|  | GAD-7 | -0.695 | 0.003^*^ |
|  | PHQ-9 | -0.559 | 0.024^*^ |
|  | BIS-II | -0.571 | 0.021^*^ |
|  | BJ-MoCA | 0.531 | 0.034^*^ |
| MA at Pz |  |  |  |
|  | Age | -0.579 | 0.019^*^ |
|  | Education | 0.734 | 0.001^*^ |
|  | BJ-MoCA | 0.883 | 0.000^**^ |
|  | RT | -0.507 | 0.045^*^ |
| MA at T5 |  |  |  |
|  | GAD-7 | -0.706 | 0.002^*^ |
|  | BJ-MoCA | 0.690 | 0.003^*^ |
|  | RT | -0.620 | 0.010^*^ |
| MA at T6 |  |  |  |
|  | Education | 0.505 | 0.046^*^ |
|  | PHQ-9 | -0.621 | 0.010^*^ |
|  | BIS-II | -0.529 | 0.035^*^ |
|  | BJ-MoCA | 0.560 | 0.024^*^ |
|  | RT | -0.539 | 0.031^*^ |
| ML at Cz |  |  |  |
|  | BJ-MoCA | -0.571 | 0.021^*^ |
|  | RT | 0.651 | 0.006^*^ |
| ML at Pz |  |  |  |
|  | BJ-MoCA | -0.514 | 0.042^*^ |
|  | RT | 0.573 | 0.020^*^ |
| ML at T5 |  |  |  |
|  | BJ-MoCA | -0.549 | 0.028^*^ |
|  | RT | 0.593 | 0.016^*^ |
| ML at T6 |  |  |  |
|  | BJ-Moca | -0.525 | 0.037^*^ |

*Note*: MA=mean amplitude, ML=mean latency, RT=response time, *p<0.05, **p<0.001. BJ-MoCA: The Beijing version of Montreal Cognitive Assessment. GAD-7: Generalized Anxiety Disorder-7. PHQ-9: Patient Health Questionnaire-9. BIS-II: Barratt Impulsiveness Scale-II. FTND: Fagerstrom Test of Nicotine Dependence.

**Table 4 Correlation analysis of** **mean amplitude and mean latency in MMT group**

| Variable | | *r* | *P* value |
| --- | --- | --- | --- |
| Dependent | Independent |  |  |
| MMT group |  |  |  |
| MA at Fz |  |  |  |
|  | Age | -0.640 | 0.010^*^ |
|  | Duration of addiction | -0.551 | 0.033^*^ |
|  | GAD-7 | -0.750 | 0.001^*^ |
|  | PHQ-9 | -0.546 | 0.035^*^ |
|  | BIS-II | -0.582 | 0.023^*^ |
|  | BJ-MoCA | 0.598 | 0.019^*^ |
|  | RT | -0.560 | 0.030^*^ |
| MA at Cz |  |  |  |
|  | Age | -0.570 | 0.027^*^ |
|  | Duration of addiction | -0.603 | 0.017^*^ |
|  | GAD-7 | -0.589 | 0.021^*^ |
|  | PHQ-9 | -0.645 | 0.009^*^ |
|  | BIS-II | -0.630 | 0.012^*^ |
|  | BJ-MoCA | 0.821 | 0.000^**^ |
|  | RT | -0.804 | 0.000^**^ |
| MA at Pz |  |  |  |
|  | GAD-7 | -0.768 | 0.001^*^ |
|  | PHQ-9 | -0.773 | 0.001^*^ |
|  | BIS-II | -0.930 | 0.000^**^ |
|  | BJ-MoCA | 0.836 | 0.000^**^ |
|  | RT | -0.826 | 0.000^**^ |
| MA at T5 |  |  |  |
|  | Age | -0.656 | 0.008^*^ |
|  | GAD-7 | -0.782 | 0.001^*^ |
|  | PHQ-9 | -0.836 | 0.000^**^ |
|  | BIS-II | -0.679 | 0.005^*^ |
|  | BJ-MoCA | 0.673 | 0.006^*^ |
| MA at T6 |  |  |  |
|  | GAD-7 | -0.571 | 0.026^*^ |
|  | PHQ-9 | -0.587 | 0.021^*^ |
| ML at Pz |  |  |  |
|  | BIS-II | 0.548 | 0.035^*^ |
| ML at T5 |  |  |  |
|  | BIS-II | 0.588 | 0.021^*^ |
|  | BJ-MoCA | -0.527 | 0.044^*^ |
|  | RT | 0.595 | 0.019^*^ |
| ML at T6 |  |  |  |
|  | BIS-II | 0.637 | 0.011^*^ |
|  | BJ-MoCA | -0.645 | 0.009^*^ |
|  | RT | 0.604 | 0.017^*^ |

*Note*: MA=mean amplitude, ML=mean latency, RT=response time, MMT=methadone maintenance treatment. *p<0.05, **p<0.001. BJ-MoCA: The Beijing version of Montreal Cognitive Assessment. GAD-7: Generalized Anxiety Disorder-7. PHQ-9: Patient Health Questionnaire-9. BIS-II: Barratt Impulsiveness Scale-II. FTND: Fagerstrom Test of Nicotine Dependence.
